# The risk of developing depression in tuberculosis survivors: a nationwide cohort study in South Korea

**DOI:** 10.3389/fpsyt.2025.1425637

**Published:** 2025-02-21

**Authors:** Hyun Soo Kim, Jin Hyung Jung, Kyungdo Han, Hyewon Kim, Hong Jin Jeon

**Affiliations:** ^1^ Department of Psychiatry, Dong-A University College of Medicine, Busan, Republic of Korea; ^2^ Department of Biostatistics, College of Medicine, Catholic University of Korea, Seoul, Republic of Korea; ^3^ Department of Statistics and Actuarial Science, Soongsil University, Seoul, Republic of Korea; ^4^ Department of Psychiatry, Hallym University Sacred Heart Hospital, Anyang, Republic of Korea; ^5^ Department of Psychiatry, Depression Center, Samsung Medical Center, Sungkyunkwan University School of Medicine, Seoul, Republic of Korea; ^6^ Department of Health Sciences & Technology, Samsung Advanced Institute for Health Sciences & Technology (SAIHST), Sungkyunkwan University, Seoul, Republic of Korea; ^7^ Department of Medical Device Management & Research, Samsung Advanced Institute for Health Sciences & Technology (SAIHST), Sungkyunkwan University, Seoul, Republic of Korea; ^8^ Department of Clinical Research Design & Evaluation, Samsung Advanced Institute for Health Sciences & Technology (SAIHST), Sungkyunkwan University, Seoul, Republic of Korea

**Keywords:** tuberculosis, gender difference, depression, anti-tuberculosis drugs, cycloserine

## Abstract

**Background:**

Despite a high tuberculosis incidence in Korea, the association between tuberculosis and depression remains underexplored. This study aims to assess depression risk in tuberculosis survivors.

**Methods:**

Utilizing South Korea’s National Health Insurance Sharing Service (NHISS) database, we conducted a gender-age-matched analysis comparing depression risk between tuberculosis survivors and the general population.

**Results:**

This study included 137,996 participants, of whom 34,499 had tuberculosis history, and 103,497 age- and sex-matched individuals were selected as the control group. The risk of developing depression was higher in tuberculosis survivors than in the control group (aHR 1.20, 95% CI 1.15-1.25). In men, the risk of developing depression was 1.32 times (95% CI 1.25-1.39) compared with 1.05 times (95% CI 0.98-1.12) in women. Those taking para-aminosalicylate, cycloserine, and prothionamide had a higher risk of developing depression compared to those using other anti-tuberculosis drugs, with the risk ratio ranging from 1.27 to 1.61.

**Conclusion:**

Tuberculosis survivors had a higher risk of developing depression compared to the control group. Although the prevalence of depression was higher in women compared to men, the risk of developing depression was higher in male tuberculosis survivors than in the control group, in contrast to the findings in women. The risk of developing depression in tuberculosis survivors differed depending on the anti-tuberculosis drug used and was mainly high in most of the second-line anti-tuberculosis drugs.

## Introduction

Depression stands as a prevalent global mental health concern, recognized as a foremost contributor to significant health challenges. Globally, more than 300 million people suffer from depression according to the World Health Organization (WHO). In Korea, about 6.7% of the population suffer from depression (1). Epidemiological surveys on mental disorders in Korea have reported a gradually increasing lifetime prevalence of depression (2).

Tuberculosis (TB) is an infectious disease caused by Mycobacterium tuberculosis bacteria. In South Korea, the incidence of TB is 80.0 per 100,000 people, which is higher than the average incidence of 11.4 in Organization for Economic Co-operation and Development (OECD) countries. The coexistence of TB and depression is a prevalent comorbidity, posing a significant challenge to public health in terms of treatment and prevention (3, 4). In particular, patients with drug-resistant tuberculosis (DR-TB) carry a substantial psychological burden, leading to the experience of depression (5). Even after the completion of TB treatment, survivors may continue to experience psychological issues such as depression and anxiety, as well as physical sequelae like COPD and lung cancer, all of which can have a long-term impact on their overall quality of life (6).

Despite the clinically significant association between TB and depression, research addressing the intersection of these two conditions is notably lacking. Thus, the purpose of this study was to compare the risk of developing depression in TB survivors with general population. We hypothesized that: 1) TB would be associated with an increased risk of developing depression; and 2) the risk of developing depression would vary according to anti-TB drugs.

## Materials and methods

### Data source

This study used the National Health Insurance Sharing Service (NHISS) database of the National Health Insurance Service (NHIS) of South Korea (7, 8). NHIS is a public institution responsible for operationalizing mandatory universal health insurance. NHISS database contains medical services claim data such as information about admission, emergency room visits, ambulatory care visits, and pharmaceutical services. These data are open to the public (https://nhiss.nhis.or.kr). The NHISS database also contains results of general health examination and questionnaires for evaluating lifestyle and behavior. NHIS data also contain sociodemographic data such as age, sex, place of residence, and income level. NHISS data are anonymized to protect the privacy of individuals. This study was approved by the Institutional Review Board of the Samsung Medical Center (SMC 2021-10-087, October 21, 2021).

### Study population

**Figure 1:**
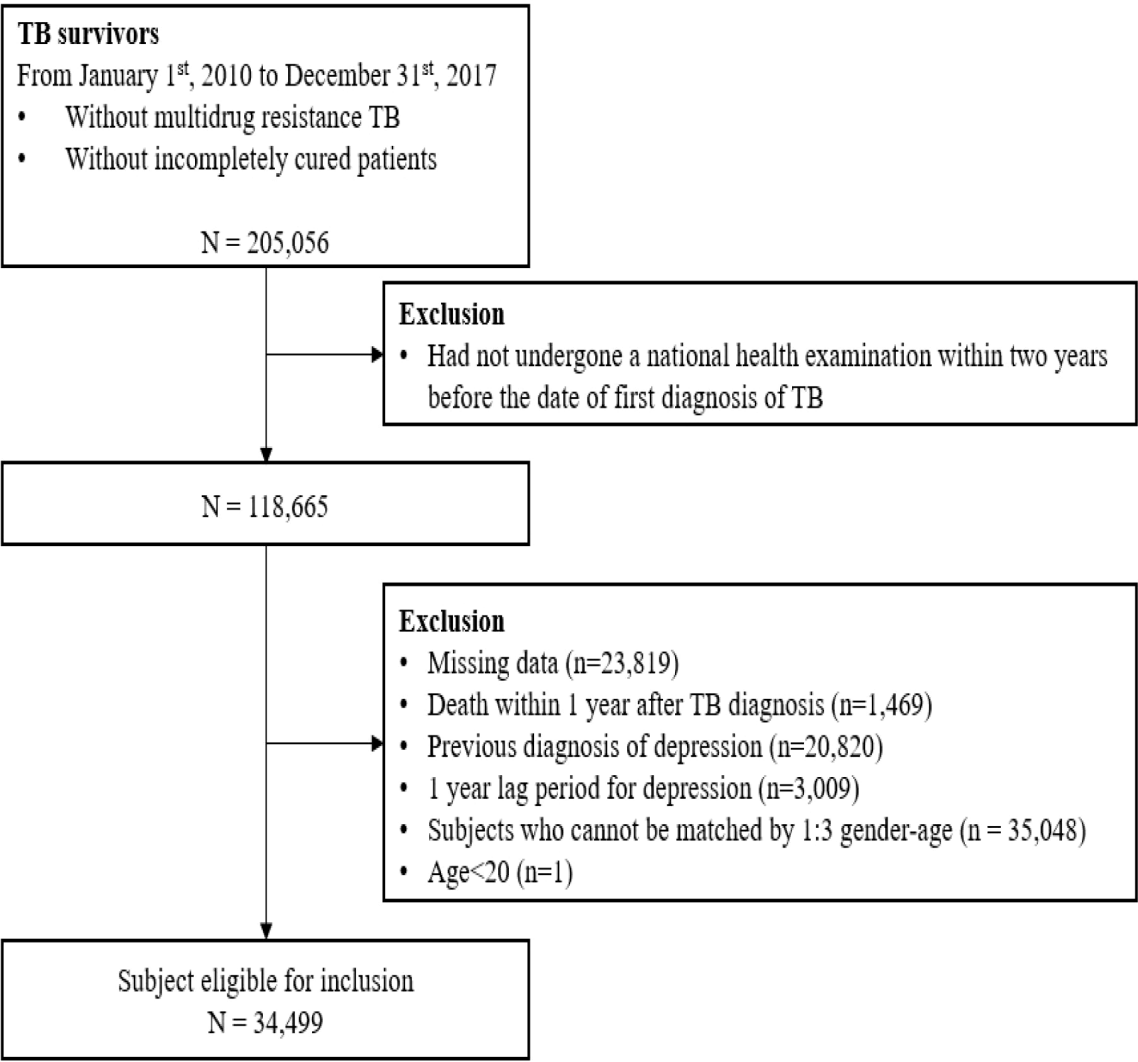
Flow chart showing the section of study population.

We characterized TB survivors as individuals aged 20 years or older who had completed treatment for active TB and lived for at least one year following their TB diagnosis. TB cases were defined as those who were initially diagnosed with International Statistical Classification of Disease and Related Health Problems 10^th^ revision (ICD-10) code “A15-A19” or with specific insurance codes of “V000, V206, V246” between January 1, 2010 and December 31, 2017. Patients with multidrug-resistant (MDR) TB (ICD-10 U88.0–U88.1) and incompletely cured patients with a treatment period of less than 156 days were excluded (9, 10). Among them, patients who visited the hospital more than twice with the relevant ICD-10 codes or TB insurance codes, or those who received at least 90 days of anti-TB treatment with drugs such as isoniazid, rifampicin, pyrazinamide, ethambutol, para-aminosalicylic acid, cycloserine, and prothionamide, were included (11). Among them, 118,665 subjects who had undergone a national health examination within two years before the date of first diagnosis of TB were included in this study. Among them, we excluded subjects with missing data (n = 23,819), those who died within one year after TB diagnosis (n = 1,469), those who had previously been diagnosed with depression (n = 20,820), and those who were diagnosed with depression within one year of TB diagnosis (n = 3,009) to avoid effects of a temporal relationship and minimized the possibility of reverse causality. Finally, 34,449 subjects aged 20 or older who could be matched for 1:3 gender-age matching were targeted. The control group included 103,497 subjects (**Figure 1**).

### Definition of study outcome

The primary outcome of this study was newly diagnosed depression defined as a diagnostic code of F32 or F33 (F32.0-32.9 for major depressive disorder with single episode and F33.0-33.9 for major depressive disorder with recurrent episodes) according to the International Statistical Classification of Disease and Related Health Problems 10th revision (ICD-10). The cohort was followed up from baseline to the date of incident of depression, death, or the end date of this study (December 31, 2018), whichever came first.

### Definition of covariates

Information about subject’s health related behaviors was obtained through self-report questionnaires. Body mass index (BMI) was calculated by dividing weight (kg) by height (m) squared and classified as low (BMI < 18.5), normal (BMI = 18.5–22.9), overweight (BMI = 23–24.9), and obesity (BMI ≥ 25). Smoking status was classified as never, former, and current smokers. Alcohol consumption was classified into non, mild to moderate (< 30 g of alcohol/day), and heavy (≥ 30 g of alcohol/day) drinkers according to the number of times and the frequency of drinking per week. Regular physical activity was defined as ≥ 30 minutes of moderate physical activity ≥ 5 times per week or ≥ 20 minutes of vigorous physical activity ≥ 3 times per week. The income level was divided based on the bottom 20% according to the monthly insurance premium because insurance contributions were determined based on income level in Korea.

Diabetes (DM) was defined as a fasting blood glucose level of 126 mg/dL or higher or diagnosed using ICD-10 codes E11-E14 and prescribed medication for DM. Hypertension (HTN) was defined as ICD-10 codes I10-I13 and I15 with antihypertensive medications or a systolic blood pressure of 140 mmHg or greater and a diastolic blood pressure of 90 mmHg or greater. Dyslipidemia was defined as a fasting serum total cholesterol of 240 mg/dL or more, diagnosed with ICD-10 code E78, and prescribed lipid-lowering agents. The Charlson Comorbidity Index was categorized as 0, 1 to 2, or ≥3 to assess the overall comorbidity load (12).

### Statistical analyses

Baseline characteristics are presented as means ± standard deviation for continuous variables and numbers (percentages) for categorical variables. We used two sample t-test or chi-square test to find differences in demographic and socioeconomic characteristics between the TB group and control group.

Kaplan-Meier curves and log-rank test were used to present and compare cumulative depression incidences for both groups. Multiple Cox proportional regression models were used to assess the risk of developing depression after adjusting for age, sex, BMI, smoking status, alcohol consumption, physical activity, income level, place of residence, DM, HTN, dyslipidemia, and CCI score as possible confounders. Results are presented as hazard ratios (HRs) with 95% confidence intervals (CIs). Model 1 was not adjusted. Model 2 was adjusted for age, sex, smoking status, alcohol consumption, regular physical activity, income level, and place of residence. Model 3 was additionally adjusted for model 2 for BMI, DM, HTN, and dyslipidemia. Model 4 was further adjusted for the CCI score. All statistical tests were two-tailed and the significance level was set at *p* < 0.05. All statistical analyses were performed using SAS version 9.4 (SAS Institute Inc., Cary, NC, USA).

## Results

### Baseline characteristics of the study subjects

A total of 137,996 people participated in this study. Of them, 34,499 people was TB survivors. A total of 103,497 people who were 1:3 matched with the TB group for age and sex were identified and included in the control group. Rates of DM and dyslipidemia were higher in the TB group than in the control group, whereas rates of HTN were higher in the control group than in the TB group. The TB group showed higher CCI score than the control group (mean ± standard deviation [SD]: 1.9 ± 1.78 vs. 1.01 ± 1.38) (**Table 1**).

**Table 1:** Baseline characteristics of study participants.

	Total Population	Matched controls	TB survivors	*p*-value
	137,996	103,497	34,499	
Sex (male)	94884 (68.76)	71163 (68.76)	23721 (68.76)	1
Age				1
20-29	5884 (4.26)	4413 (4.26)	1471 (4.26)	
30-39	17852 (12.94)	13389 (12.94)	4463 (12.94)	
40-49	34052 (24.68)	25539 (24.68)	8513 (24.68)	
50-59	38564 (27.95)	28923 (27.95)	9641 (27.95)	
60-69	26260 (19.03)	19695 (19.03)	6565 (19.03)	
70-79	14536 (10.53)	10902 (10.53)	3634 (10.53)	
≥80	848 (0.61)	636 (0.61)	212 (0.61)	
Body Mass Index (BMI)				<.0001
<18.5	6170 (4.47)	2882 (2.78)	3288 (9.53)	
18-22.9	54719 (39.65)	36372 (35.14)	18347 (53.18)	
23-24.9	34059 (24.68)	26972 (26.06)	7087 (20.54)	
>=25	43048 (31.20)	37271 (36.01)	5777 (16.75)	
Place of residence	62485 (45.28)	46213 (44.65)	16272 (47.17)	<.0001
Smoking status				<.0001
Never smoker	68934 (49.95)	52764 (50.98)	16170 (46.87)	
Ex-smoker	29373 (21.29)	23029 (22.25)	6344 (18.39)	
Current smoker	39689 (28.76)	27704 (26.77)	11985 (34.74)	
Alcohol consumption				<.0001
None	64272 (46.58)	47905 (46.29)	16367 (47.44)	
Mild to moderate	60476 (43.82)	46626 (45.05)	13850 (40.15)	
Heavy	13248 (9.60)	8966 (8.66)	4282 (12.41)	
Regular physical activity	28508 (20.66)	22399 (21.64)	6109 (17.71)	<.0001
Income level (low)	22839 (16.55)	16714 (16.15)	6125 (17.75)	<.0001
Charlson comorbidity index	1.23 ± 1.54	1.01 ± 1.38	1.9 ± 1.78	<.0001
0	60540 (43.87)	52028 (50.27)	8512 (24.67)	
1	32263 (23.38)	23640 (22.84)	8623 (24.99)	
2	20708 (15.01)	13868 (13.4)	6840 (19.83)	
≥3	24485 (17.74)	13961 (13.49)	10524 (30.51)	
Comorbidity				
Diabetes	18695 (13.55)	12373 (11.95)	6322 (18.33)	<.0001
Hypertension	34288 (24.85)	26152 (25.27)	8136 (23.58)	<.0001
Dyslipidemia	31032 (22.49)	22429 (21.67)	8603 (24.94)	<.0001

### Hazard ratios of tuberculosis for developing depression

**Figure 2:**
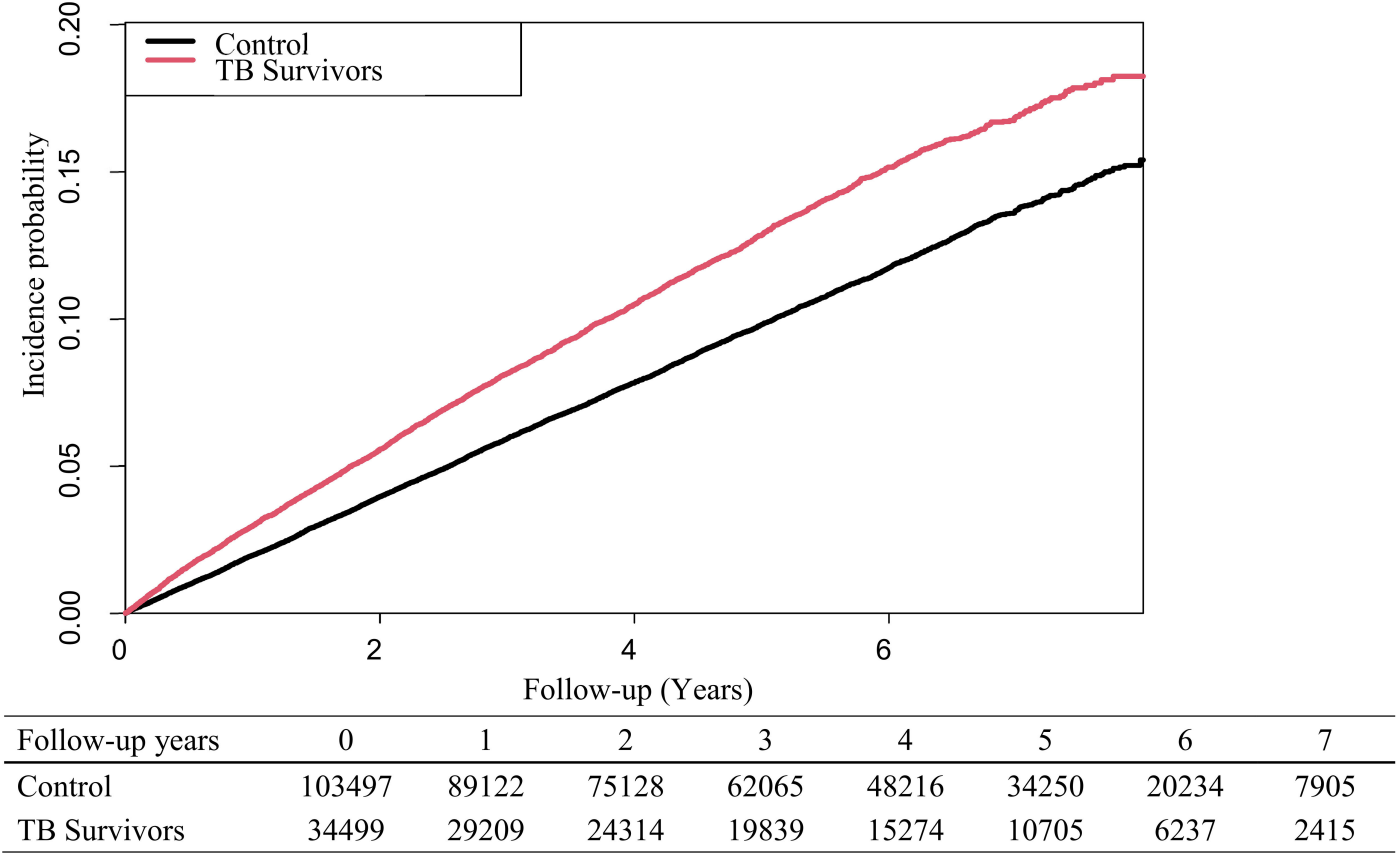
Kaplan-Meier curves for the risk of developing depression in tuberculosis survivors and matched controls.

Overall, the risk of developing depression was higher in TB survivors than in the control group (adjusted hazard ratio [aHR]: 1.20, 95% CI: 1.15-1.25). In men, the risk of developing depression was 1.32 times (95% CI: 1.25-1.39) higher in the TB group than in the control group. However, for women, the TB group did not show significantly increased risk of developing depression than in the control group (**Table 2**). **Figure 2** presents Kaplan- Meier curves for cumulative incidence probability of depression according to TB during the follow-up period. Results revealed a significant difference in cumulative incidence probability between TB survivors and matched controls (log-rank *p* < 0.001).

**Table 2:** Hazard ratios and 95% confidence intervals for the incidence of developing depression in tuberculosis survivors compared with matched controls.

Subgroup		Group	Subjects (n)	Depression (n)	Duration (person-years)	Incidence rate (per 1000)	Hazard Ratio (95% CI)			
							Model 1	Model 2	Model 3	Model 4
Overall		Control	103497	8026	388620.7	20.65	1 (Ref.)	1 (Ref.)	1 (Ref.)	1 (Ref.)
		TB	34499	3445	125100.8	27.54	1.33 (1.28,1.39)	1.34 (1.29,1.39)	1.29 (1.23,1.34)	1.2 (1.15,1.25)
Sex	Men	Control	71163	4648	265828.4	17.49	1 (Ref.)	1 (Ref.)	1 (Ref.)	1 (Ref.)
		TB	23721	2182	85036.2	25.66	1.47 (1.40,1.55)	1.46 (1.39,1.54)	1.46 (1.39,1.54)	1.25 (1.18,1.32)
	Women	Control	32334	3378	122792.3	27.51	1 (Ref.)	1 (Ref.)	1 (Ref.)	1 (Ref.)
		TB	10778	1263	40064.6	31.52	1.15 (1.07,1.22)	1.17 (1.09,1.25)	1.17 (1.09,1.25)	1.02 (0.95,1.09)

Model 1 was not adjusted.

Model 2 was adjusted for age, sex, smoking, alcohol consumption, regular physical activity, income level, and place of residence.

Model 3 was adjusted for age, sex, smoking, alcohol consumption, regular physical activity, income level, place of residence, BMI, DM, HTN, and dyslipidemia.

Model 4 was adjusted for age, age, sex, BMI, smoking, alcohol consumption, regular physical activity, income level, place of residence, BMI, DM, HTN, dyslipidemia, and CCI score.

TB; tuberculosis; BMI, body mass index; DM, diabetes; HTN, hypertension; CCI, Charlson comorbidity index.

### Hazard ratios of anti-tuberculosis drugs for developing depression

Among TB survivors, those who took isoniazid, rifampicin, and ethambutol had lower risks of developing depression compared to those who did not take these drugs. In the case of taking pyrazinamide, the hazard ratio of developing depression was 1.27 (95% CI: 1.18-1.37), which was higher than that (1.18, 95% CI: 1.13-1.24) in the group of TB survivors who did not take pyrazinamide. For TB survivors taking para-aminosalicylate, cycloserine, and prothionamide, hazard ratios of developing depression were 1.52 (95% CI: 1.11-2.09), 1.52 (95% CI: 1.28-1.82), and 1.61 (95% CI: 1.29-2.00), respectively, compared to the control group. These were higher than the risk of TB survivors who did not take these drugs (**Table 3**).

**Table 3:** Hazard ratios and 95% confidence intervals for the incidence of developing depression in tuberculosis survivors according to anti tuberculosis drugs.

Anti-Tuberculosis drug	Group	Subjects (n)	Depression (n)	Duration (person-years)	Incidence rate (per 1000)	Hazard Ratio (95% CI)			
						Model 1	Model 2	Model 3	Model 4
Isoniazid	Control	103497	8026	388620.71	20.65	1(Ref.)	1(Ref.)	1(Ref.)	1(Ref.)
	Non user with TB	1906	209	6612.31	31.69	1.53(1.34,1.76)	1.44(1.26,1.65)	1.38(1.20,1.58)	1.28(1.12,1.47)
	User with TB	32593	3236	118488.48	27.31	1.32(1.27,1.38)	1.33(1.28,1.39)	1.28(1.23,1.34)	1.20(1.15,1.25)
Rifampicin	Control	103497	8026	388620.71	20.65	1(Ref.)	1(Ref.)	1(Ref.)	1(Ref.)
	Non user with TB	1231	139	4310.94	32.24	1.57(1.32,1.85)	1.53(1.30,1.81)	1.45(1.22,1.71)	1.34(1.13,1.58)
	User with TB	33268	3306	120789.85	27.37	1.33(1.27,1.38)	1.33(1.28,1.39)	1.28(1.23,1.34)	1.20(1.15,1.25)
Pyrazinamide	Control	103497	8026	388620.71	20.65	1(Ref.)	1(Ref.)	1(Ref.)	1(Ref.)
	Non user with TB	27211	2673	98568.33	27.12	1.31(1.26,1.37)	1.32(1.26,1.38)	1.27(1.21,1.33)	1.18(1.13,1.24)
	User with TB	7288	772	26532.46	29.10	1.41(1.31,1.52)	1.42(1.32,1.53)	1.36(1.26,1.47)	1.27(1.18,1.37)
Ethambutol	Control	103497	8026	388620.71	20.65	1(Ref.)	1(Ref.)	1(Ref.)	1(Ref.)
	Non user with TB	5050	490	15624.86	31.36	1.52(1.39,1.67)	1.42(1.30,1.56)	1.36(1.24,1.49)	1.26(1.15,1.38)
	User with TB	29449	2955	109475.93	26.99	1.31(1.25,1.36)	1.32(1.27,1.38)	1.28(1.22,1.33)	1.19(1.14,1.25)
Para-aminosalicylate	Control	103497	8026	388620.71	20.65	1(Ref.)	1(Ref.)	1(Ref.)	1(Ref.)
	Non user with TB	34218	3407	123889.56	27.50	1.33(1.28,1.39)	1.33(1.28,1.39)	1.28(1.23,1.34)	1.20(1.15,1.25)
	User with TB	281	38	1211.24	31.37	1.52(1.11,2.09)	1.72(1.25,2.37)	1.63(1.19,2.25)	1.52(1.11,2.09)
Cycloserine	Control	103497	8026	388620.71	20.65	1(Ref.)	1(Ref.)	1(Ref.)	1(Ref.)
	Non user with TB	33432	3316	121171.82	27.37	1.33(1.27,1.38)	1.33(1.27,1.38)	1.28(1.22,1.33)	1.19(1.14,1.25)
	User with TB	1067	129	3929.98	32.83	1.60(1.34,1.90)	1.71(1.44,2.04)	1.63(1.37,1.94)	1.52(1.28,1.82)
Prothionamide	Control	103497	8026	388620.71	20.65	1(Ref.)	1(Ref.)	1(Ref.)	1(Ref.)
	Non user with TB	33828	3365	122606.02	27.45	1.33(1.28,1.38)	1.33(1.28,1.38)	1.28(1.23,1.33)	1.20(1.15,1.25)
	User with TB	671	80	2494.77	32.07	1.56(1.25,1.94)	1.79(1.44,2.23)	1.71(1.37,2.13)	1.61(1.29,2.00)

Model 1 was not adjusted.

Model 2 was adjusted for age, sex, smoking, alcohol consumption, regular physical activity, income level, and place of residence.

Model 3 was adjusted for age, sex, smoking, alcohol consumption, regular physical activity, income level, place of residence, BMI, DM, HTN, and dyslipidemia.

Model 4 was adjusted for age, age, sex, BMI, smoking, alcohol consumption, regular physical activity, income level, place of residence, BMI, DM, HTN, dyslipidemia, and CCI score.

TB; tuberculosis; BMI, body mass index; DM, diabetes; HTN, hypertension; CCI, Charlson comorbidity index.

## Discussion

This study was about the relationship between TB and depression using data from the NHIS database of Korea. We hypothesized that: 1) TB would be associated with an increased risk of developing depression; and 2) the risk of developing depression would vary according to anti-TB drugs. Considering the results of this study, the first hypothesis was confirmed to have a high risk of developing depression in TB survivors. And the second hypothesis was confirmed through the finding that patients taking second-line anti-TB drugs such as para-aminosalicylate, cycloserine, and prothionamide had a higher risk of developing depression.

Previous studies have reported that not only patients with TB, but also individuals who have completed TB treatment, exhibit a higher incidence of depression and mental disorders (13, 14). A retrospective cohort study reported that individuals with a history of TB have a 24% higher risk of developing depression compared to those without a history of TB (13). Results of this study were similar to those of previous study in that TB survivors had a higher risk of developing depression than the general population. The increased risk of developing depression in patients with TB history is attributable to various biopsychosocial aspects. From a biological point of view, immuno-inflammatory response and lipid metabolism are considered potential mechanisms in the relationship between depression and TB (4). Human immune deficiency virus infection, poor social support, and perceived stigma can increase the risk of developing depression among patients with TB (15). TB infection or reactivation may precipitate depression, likely as a consequence of host’s inflammatory response and/or dysregulation of the hypothalamic-pituitary-adrenal axis (4). Considering social aspects, economic difficulties and malnutrition in patients with TB are risk factors for depression (3).

In this study, the risk of developing depression was significant in men with TB history, but not in women, compared to matched controls. A previous review article has reported that women with TB have a higher risk of developing depression than men (14).Some studies have suggested that the risk of developing depression is higher in men than in women among individuals with a history of TB, while most previous studies on gender differences in depression among patients with TB have indicated a higher risk for women (13). However, the present study found that the risk of developing depression was higher in men. The results of this study may differ from those of other studies, as depression was assessed during the follow-up period after the completion of TB treatment. In addition, this difference might be that unlike previous studies that primarily compared males and females, the present study distinguished itself by comparing the TB population with the general population. In this respect, this study was different from previous studies in that it excluded the risk of gender itself for developing depression and only compared synergistic effect of TB between men and women.

Worldwide, TB prevalence and active TB occurrence are higher in men than in women (16, 17). A study on gender differences of TB in Korea has reported that the mortality rate due to TB is higher in men than in women (18). A cohort study conducted in Australia and Taiwan has found that the prognosis of TB is worse and more severe in men than in women (19, 20). When considering the combined findings of these research results, it becomes evident that TB imposes a greater burden on men than on women (21). These gender differences in the disease course of TB can be considered as a cause of the synergistic effect of TB-induced depression in men.

Nevertheless, when considering the overall prevalence of depression, the rate of depression in women was found to be higher than in both the men with TB group and the control group. While the impact of TB on the development of depression is more pronounced in men than in women, the overall risk of developing depression remains higher in women. This suggests that the influence of gender on depression incidence may outweigh the effect of TB itself. Previous studies on gender and depression have indicated that women are approximately twice as likely to develop depression compared to men (22). A study conducted in South Korea also reported that women have a 1.58 times higher risk of developing depression than men (23). Therefore, although the increased risk of depression due to TB is higher in men than in women, attention to depression in women should be continuously maintained, as the prevalence of depression is higher in women.

Result of this study revealed that second-line anti-TB drugs such as para-aminosalicylate, cycloserine, and prothionamide increased the risk of developing depression (10, 24). Second-line anti-TB drugs, known for low tolerance and side effects, may include depression as a potential side effect (24). In a study conducted in Iran, it was reported that among the adverse effects of drugs used for MDR-TB, neurological side effects such as depression and suicide accounted for a large proportion (25). Among these second-line anti-TB drugs, especially, cycloserine has been reported to have neuropsychiatric toxicity, including depression, psychosis, and neuropathy due to good CSF penetration (26, 27). Severe psychiatric symptoms such as hallucinations, anxiety, and depression have been reported in 9.7%-50% of the patient group taking cycloserine (28). However, considering that most drugs mainly used as secondary anti-TB drugs have been shown to increase the risk of developing depression, other causes besides causes of individual drugs can be considered.

Patients with treatment-resistant TB had a high psychological burden due to effects of long-term TB treatment and chronic nature of MDR-TB, which can lead to a high risk of developing anxiety and depressive disorder (5). Patients’ feelings of isolation and hopelessness over long-term TB treatment using anti-TB drugs can contribute to the formation of depression (29). Second-line anti-TB drugs for TB are mainly used when first-line anti-TB drugs cannot be used due to low tolerance and side effects (24). Although MDR-TB patients were excluded from this study, the increased risk of developing depression among those who used para-aminosalicylate, cycloserine, and prothionamide suggests that the risk of depression could increase when drug treatment for TB is ineffective, a situation similar to MDR-TB. Also, given that depression can reduce TB treatment compliance, second-line anti-drugs might have been used due to low treatment compliance caused by undiagnosed depression (3).

In this study, it was found that cycloserine increased depression in TB group. However, some studies have shown that cycloserine is helpful for treating other psychiatric diseases including depression (30, 31). Cycloserine has been studied as a treatment for various neuropsychiatric disorders (such as schizophrenia, anxiety disorders, addiction, eating disorders, depression, and autism) and TB (30). In addition, there is also a research that taking cycloserine increases the therapeutic effect of cognitive behavior therapy, which is used for the treatment of anxiety disorders (31). Pharmacologically, cycloserine is a partial agonist for N-methyl-D-aspartate (NMDA). However, at high doses, it has an NMDA antagonistic action (32, 33). NMDA antagonists have antidepressant effects, which are helpful in the treatment of depression and acute suicidal ideation (34). Although results of this study showed that cycloserine increased the risk of depression, considering previous studies on cycloserine and depression, future studies need to determine whether cycloserine should be used as a treatment for depression in patients with TB.

Treating the accompanying depression is also an important part of TB management. Untreated depression can lead to persistent functional decline and a reduction in quality of life, even after TB treatment (35). In addition, this is because if depression accompanying in patients with TB is not treated, it may adversely affect results of TB treatment (36). Depression can lower treatment adherence in TB by affecting hygiene activities along with pathogenic mechanisms (3). Therefore, depression treatment is considered to play a very important role in the treatment of TB. It is necessary to take an approach by viewing TB and depression as one problem, not separate problems.

Appropriate antidepressant selection is important in the treatment of TB patients with depression. Anti-TB drugs, especially rifampicin, isoniazid, and fluoroquinolone, show clinically significant drug interactions with co-administered drugs during TB treatment. Thus, clinical caution is needed when using them (37). For this reason, it is important to manage drug-drug interactions with anti-TB drugs to improve the cure rate of TB and the quality of life of patients with TB (38). Drug-drug interactions of most anti-TB drugs are related to cytochrome P450 isoenzymes. Selective serotonin reuptake inhibitor, an antidepressant mainly used by clinicians, is also metabolized by cytochrome P450 isoenzymes (37, 39). Desvenlafaxine, the primary metabolite of venlafaxine, has a relatively simple metabolic pathway compared to other antidepressants. Thus, it has fewer interactions with drugs used for a variety of other physical conditions (39). Comparison of the safety of antidepressants including desvenlafaxine with anti-TB drugs in depressed patients with TB could help clinicians select antidepressants.

This study has several limitations. First, the diagnosis of depression and TB was based on ICD-10 codes and prescription history, which means that patients with undiagnosed TB or depression were excluded. In particular, individuals who had depression before the onset of TB but were neither diagnosed nor treated for it would have been classified as not having depression in the past. As a result, some patients with a history of depression may have been misclassified as having newly diagnosed depression. Furthermore, while depression onset may be related to TB, other factors such as comorbid conditions, psychosocial factors, and other medications could also influence depression development. Unfortunately, these factors could not be fully accounted for in this study. Second, the self-reported nature of the health examination questionnaire asking about personal life styles could be affected by recall bias. Third, since those who had participated in the general health screening program were included in the analyses, there was a potential for selection bias. Fourth, this study only targeted Koreans with a relatively high incidence of TB, making it difficult to generalize results to other ethnicities and countries. Fifth, not all anti-TB drugs were evaluated. In addition, the number of subjects used for certain anti-TB drugs was relatively small. In future studies, it would be helpful to add other anti-TB drugs, including injections, and analyze them according to first-line and second-line anti-TB drugs. Sixth, the limited follow-up period of this study may have resulted in undiagnosed cases of depression that occurred after the study period. Additionally, since the TB treatment was completed, there may have been a lack of medical need for further care, which could have led to the underdiagnosis and undertreatment of depression.

Despite these limitations, this study was meaningful in that it investigated the relationship between TB and depression through a large cohort in Korea. It was meaningful in that the risk of developing depression due to TB itself and anti-TB drugs was determined by comparing TB survivors and age and sex matched controls. Future research should aim to further investigate the gender differences in the mechanisms through which TB may contribute to the development of depression. Additionally, more definitive studies are needed to clarify the relationship between cycloserine and depression. Given the significant burden of depression among TB survivors, future studies should focus on exploring more comprehensive treatment options and strategies that not only address physical health but also provide better support for the mental health needs of TB survivors experiencing depression (6).

## Conclusions

This study investigated the risk of developing depression in TB survivors using a large cohort in Korea. TB survivors had a higher risk of developing depression. In particular, the increased risk of depression was more prominent in men than in women. The risk of developing depression in TB survivors differed depending on the anti-TB drug used. It was high mainly in those who took second-line anti-TB drugs.

## Data Availability

The raw data supporting the conclusions of this article will be made available by the authors, without undue reservation.
